# Neutralizing SARS-CoV-2

**DOI:** 10.7554/eLife.64496

**Published:** 2020-12-15

**Authors:** Eric Poeschla

**Affiliations:** Division of Infectious Diseases, University of Colorado School of Medicine, Anschutz Medical CampusAuroraUnited States

**Keywords:** COVID-19, SARS-CoV-2, VSV, antibody, viruses, Virus

## Abstract

Experiments with hybrid viruses are illuminating how SARS-CoV-2 can escape neutralizing antibodies.

**Related research article** Weisblum Y, Schmidt F, Zhang F, DaSilva J, Poston D, Lorenzi JC, Muecksch F, Rutkowska M, Hoffmann HH, Michailidis E, Gaebler C, Agudelo M, Cho A, Wang Z, Gazumyan A, Cipolla M, Luchsinger L, Hillyer CD, Caskey M, Robbiani DF, Rice CM, Nussenzweig MC, Hatziioannou T, Bieniasz PD. 2020. Escape from neutralizing antibodies by SARS-CoV-2 spike protein variants. *eLife* **9**:e61312. doi: 10.7554/eLife.61312

November brought a weary world ample reasons for celebration, even as COVID-19 cases surged anew. Among the welcome tidings were reports that mRNA-based vaccines directed at the SARS-CoV-2 spike protein may have efficacies as high as 95%, including in the elderly and other important sub-populations (Jackson et al., 2020; Sahin et al., 2020; Walsh et al., 2020; Widge et al., 2020). In addition, there are early though less definitive suggestions that monoclonal antibodies – antibodies of single specificity generated by cloning and immortalizing a plasma B cell – that target the spike protein may be therapeutic if given early (Chen et al., 2020; Rogers et al., 2020), which is a welcome counterpoint to the generally disappointing results with convalescent plasma (that is, plasma from patients who have recovered from COVID-19; Simonovich et al., 2020). Much now depends upon understanding the human neutralizing antibody response to SARS-CoV-2.

One shortcoming of convalescent plasma is that the levels of neutralizing antibodies are extremely variable, and frequently very low (Muecksch et al., 2020), with higher levels of both immunoglobulin G and immunoglobulin A correlating with more severe disease (Cervia et al., 2020). Levels also decline rapidly, by more than 50% in the first three months (Muecksch et al., 2020; Seow et al., 2020). On the other hand, monoclonals with potent neutralization capacity have been consistently obtainable from recovered COVID-19 patients and the relatively low levels of somatic hypermutation – the process by which B cells optimize antibody affinity – observed in these antibodies suggests that they might be readily elicited with the right vaccine (Robbiani et al., 2020; Yuan et al., 2020). However, it is important to understand the probability that SARS-CoV-2 may evolve to escape neutralizing antibodies, whether they are natural, vaccine-induced, or administered monoclonals.

Now, in eLife, Theodora Hatziioannou, Paul Bieniasz and co-workers – including Yiska Weisblum and Fabian Schmidt, both of Rockefeller University, as joint first authors – report data that are timely and important in this context (Weisblum et al., 2020). The researchers performed experiments in which human cells were infected, in the presence of antibodies, with a hybrid virus that mimics SARS-CoV-2. The only virus particles that could survive to propagate onward were those that had mutated in a way that allowed them to escape the antibodies. Specifically, the envelope glycoprotein of an innocuous rabies family virus was substituted with the SARS-CoV-2 spike protein (Figure 1). The antibody neutralization sensitivity of this chimeric virus tracks remarkably close to that of SARS-CoV-2, and it also provides a number of additional advantages: it enables high-throughput analyses without requiring high levels of biosecurity; it can be monitored by GFP fluorescence; and it enables the rapid selection of escape mutants because the virus propagates to high titers and – unlike a coronavirus – does not proofread mistakes made during genome copying.

**Figure 1.**
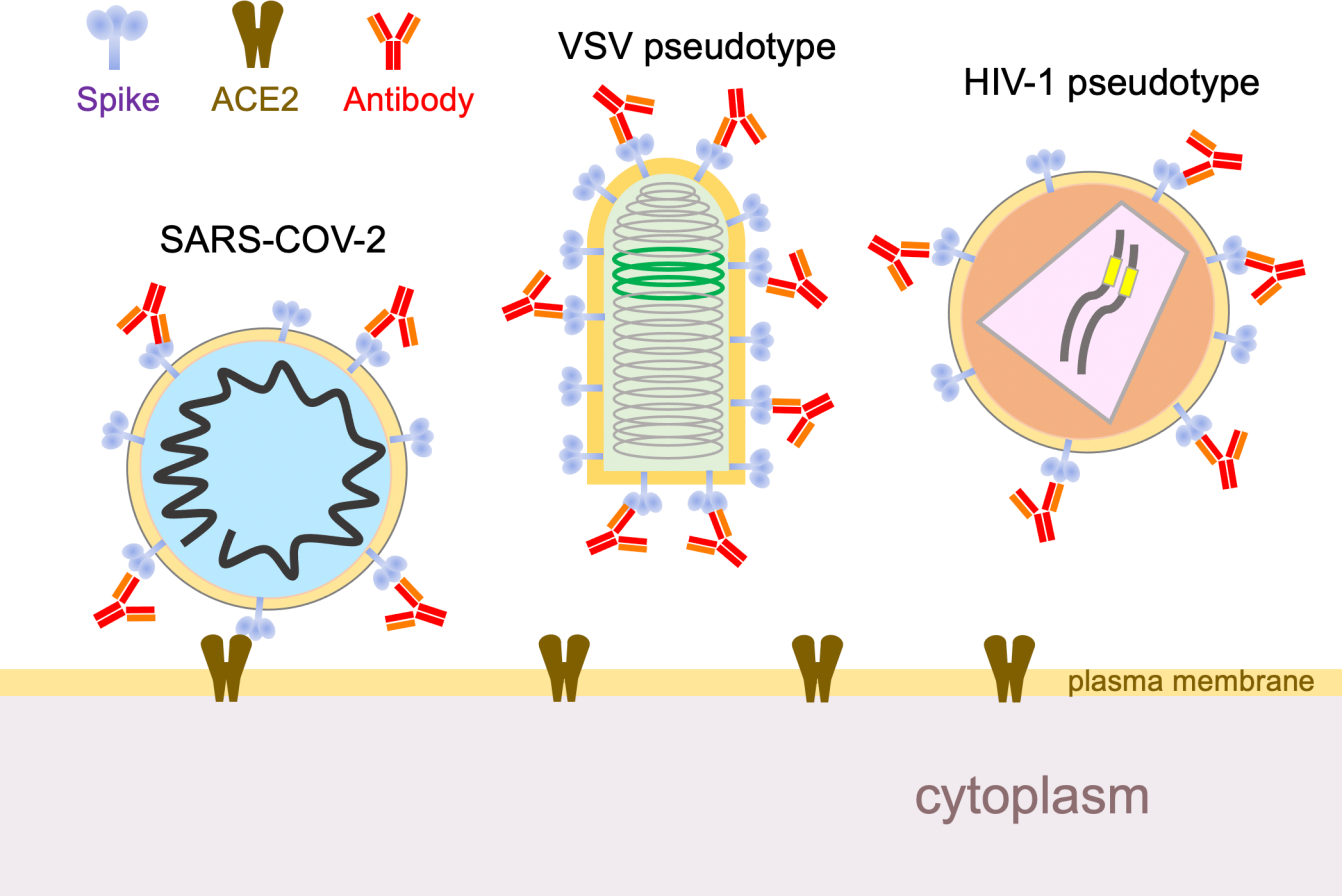
Using hybrid viruses to study SARS-CoV-2 escape from neutralizing antibodies. The surface of the SARS-CoV-2 virion (left) contains spike proteins (pale blue) that bind to ACE2 receptors (brown), which leads to membrane fusion and entry into the cell. Neutralizing antibodies (red) can stop this happening by binding to the spike proteins, so viruses undergo reciprocal evolution to escape such antibodies. To better understand how viruses evolve to become resistant to different kinds of antibodies, Weisblum et al. developed two hybrid viruses that could be studied in the laboratory. The first was a hybrid rabies family virus (VSV, middle) that carries the SARS-CoV-2 spike protein rather than the normal envelope protein in its outer lipid envelope. This hybrid is replication-competent, carries a GFP transgene (green), and can be used for experiments in which it undergoes serial passage and selection in the presence of convalescent plasma or monoclonal antibodies. The second hybrid was an HIV-1 vector pseudotyped with the spike protein. This hybrid is replication-defective, carries a luciferase transgene (yellow), and completes a single cycle of infection. VSV: vesicular stomatitis virus.

In the presence of potent monoclonal antibodies that target the receptor binding domain of the spike protein, and some but not all convalescent plasmas, the researchers found that it took only two or three passages to select for specific resistance. (An excellent physical feel for these experiments can be had by looking at figure 1B in Weisblum et al., 2020 at higher magnification). When the escaped viruses were sequenced, mutations in the receptor binding domain – and some outside it as well – were identified. None of these mutations impaired replicative fitness in cultured cells in a discernible way.

Notably, mutations that potently blocked a given monoclonal antibody conferred little or no resistance to neutralization by plasma from the same individual (or others). Conversely, plasma from a given individual did not select for resistance to monoclonal antibodies derived from that individual. Finally, there was no overlap in the resistance selected by convalescent plasma from different individuals, suggesting that humoral immune responses are significantly heterogeneous between individuals. However, Weisblum et al. point out that they mostly tested immunoglobulin G in their experiments, whereas immunoglobulin A predominates in lung secretions and on the surfaces of respiratory epithelia, and may be particularly beneficial in the case of SARS-CoV-2 (Wang et al., 2020).

Weisblum et al. then asked an important question: do these mutant viruses exist in real SARS-CoV-2 in the general human population? For these experiments, they used a non-replicating hybrid virus, one based on HIV-1 (Figure 1). Moreover, in addition to the escape mutants selected in their first set of experiments, they used this system to test numerous naturally occurring mutations that have been identified in or near the ACE2 binding site (http://cov-glue.cvr.gla.ac.uk/#/home). This enabled them to identify additional escape mutants. Moreover, both sets of mutants are present, albeit at very low frequencies, in naturally circulating SARS-CoV-2. Thus, they 'pre-exist' and are available for selection to prominence under specific humoral immune pressure.

This situation brings to mind a lesson well learned about RNA viruses from HIV-1, which generates exceptional diversity. Not only is resistance to antiretroviral therapy quickly induced unless combinations of drugs are used, the virus in any one patient is virtually always resistant to the antibodies present in contemporaneous plasma. The problem is less severe for coronaviruses which, alone among RNA viruses, have the ability to proofread errors made during genome copying. Even so, it is likely that all possible single amino acid variants of SARS-CoV-2 currently exist many times over in the global population and perhaps even in many infected individuals. Following the paradigm of antiretroviral therapy, we can anticipate that combinations of therapeutic monoclonal antibodies will be required to securely prevent SARS-CoV-2 resistance. Indeed, Weisblum et al. go on to show that combinations of two antibodies can block the generation of resistance in vitro.

Whether antibody escape will become clinically significant for therapeutics or vaccines is not yet clear and depends on many factors, including the frequency of reinfection – which clearly happens with the four seasonal coronaviruses – and the duration of antibody responses after natural and vaccine-induced immunity. At present, however, there is no evidence that functionally significant SARS-CoV-2 variants have emerged as a result of immune pressure.

Finally, most patients – even those with low aggregate neutralizing activity in plasma – were found to have the ability to generate potent antibodies at low levels. Moreover, the genes of the potent monoclonal antibodies identified so far differ little from the germline sequence (Robbiani et al., 2020; Yuan et al., 2020). It thus seems likely that a properly designed vaccine can induce a durable and potent neutralizing antibody response, which is likely to be more effective and longer-lasting – and much more safely acquired – than responses that follow natural infection. The early mRNA vaccine clinical trial data certainly support initial effectiveness, with duration of immunity the major outstanding question to be answered.

One of the distinctive pleasures of being a virologist is the ability to see the most important and powerful idea in biology – natural selection – happen in real time. It’s even better when the experiments yield useful insights into an urgent medical problem. In this regard, Weisblum et al. have not disappointed.
